# Effects of Autologous Platelet-Rich Plasma in women with repeated implantation failure undergoing assisted reproduction

**DOI:** 10.5935/1518-0557.20210046

**Published:** 2022

**Authors:** Afsaneh Shah Bakhsh, Narges Maleki, Mohammad Reza Sadeghi, Ali SadeghiTabar, Maryam Tavakoli, Simin Zafardoust, Atousa Karimi, Sonai Askari, Sheyda Jouhari, Afsaneh Mohammadzadeh

**Affiliations:** 1 Avicenna Fertility Clinic, Avicenna Research Institute, ACECR, Tehran, Iran; 2 Gynecology and reproductive biology Department, Kowsar polyclinic, Imam Khomeini square, Pardis Town, Tehran Province, Iran; 3 Reproductive Biotechnology Research Center, Avicenna Research Institute, ACECR, Tehran, Iran; 4 Monoclonal Antibody Research Center, Avicenna Research Institute, ACECR, Tehran, Iran; 5 Nanobiotechnology Research Center, Avicenna Research Institute, ACECR, Tehran, Iran

**Keywords:** recurrent implantation failure, PRP, platelet-rich plasma, intracytoplasmic injection

## Abstract

**Objective:**

Repeated implantation failure (RIF) is a major challenge in reproductive medicine. On the other hand, there has not yet been established a confirmed outcome regarding the usage of platelet-rich plasma (PRP) in women undergoing intracytoplasmic injection (ICSI) or in-vitro fertilization (IVF); hence, the objective of this study was to evaluate the effect of the intrauterine infusion of PRP on pregnancy outcomes in women undergoing ICSI.

**Methods:**

In this prospective double-blind clinical trial, 100 women with at least two previous unexplained RIF, who were candidates for frozen-thawed embryo transfer, were allocated into two groups. One subgroup of patients was treated by intrauterine infusion of PRP (0.5CC, contained platelet 4-5 times more than a peripheral blood sample, which was performed 48 hours before blastocyst transfer) and the other subgroup was treated by intrauterine catheterization only. We compared the implantation rates between the two groups.

**Results:**

The pregnancy rate was 20% in the intervention subgroup, while in the control subgroup it was 13.33%; therefore, there was a significant statistical difference between the two groups.

**Conclusions:**

According to this paper, PRP could be successful in improving the pregnancy outcome in RIF patients, and we highly recommend other studies with larger samples to confirm the PRP therapy efficacy in RIF patients.

## INTRODUCTION

Recurrent implantation failure (RIF) is defined as the failure to conceive following numerous embryo transfers (ET) - embryo placement in the uterus, in assisted fertilization cycles, or the absence of a gestational sac upon ultrasound at five weeks or more after ET following 3 ET with high-quality embryos, or subsequent to transferring ten or more embryos in multiple transfers, although we still lack standard criteria for RIF description. Implantation rate (IR) was defined as the number of gestational sacs seen upon echo-graphic screening at 6 weeks of pregnancy, divided by the number of embryos transferred. Pregnancy rate is the success rate for getting pregnant. The percentage of all attempts leads to pregnancy. An ongoing pregnancy is defined as each pregnancy with a positive heart beat at ultrasound after 12 weeks of gestation (Coughlan *et al*., 2014; Simon & Laufer, 2012; El-Toukhy & Taranissi, 2006; Greco *et al*., 2014; Salehpour *et al*., 2016). RIF could be classified into four main subcategories: poor endometrial receptivity, embryonic defects, abnormal embryo-endometrial inter-communication, and immunologic impairment (Diedrich *et al*., 2007). Since too many patients are suffering from RIF, various clinical interventions have been utilized to increase implantation rates and subsequently decrease the financial and emotional burden on the healthcare system, as well as the affected couples, namely fixing defects in the cavity, preimplantation genetic screening (PGT-a), use of immunomodulators, assisted hatching (Ferrero *et al*., 2005), sequential transfer, endometrial scratching and stimulation, endometrial receptivity array (ERA) (Gibreel *et al*., 2015; Paulson, 2011), blastocysts and cytoplasmic transfer (Glujovsky *et al*., 2016; Barritt *et al*., 2001). In these regards, PRP was administered lately.

PRP is a concentration of protein derived from fresh whole blood, free from red blood cells, containing several cytokines as well as a large group of growth factors such as interleukin 8 (IL-8), insulin-like growth factor I, II (IGF-I, II), vascular endothelial growth factor (VGEF), platelet-derived growth factor (PDGF), fibroblast growth factor(FGF), transforming growth factor (TGF), and connective tissue growth factor (CTGF); and it is assumed to enhance endometrial growth and receptivity. The regulatory effect of PRP on the expression of growth factors and cytokines in the endometrium is due to its anti-inflammatory and pro-regenerative functions (Bos-Mikich *et al*., 2018; Chang *et al*., 2015; Garcia-Velasco *et al*., 2016; Rossi *et al*., 2017).

Although PRP is used widely in other fields of medicine, its clinical efficacy in the field of obstetrics and gynecology is still very limited. In this study, we aimed to investigate the effects of intrauterine infusion of PRP in women suffering from RIF undergoing ICSI cycles, because the results of previous studies are not all in favor of PRP application (Eftekhar *et al*., 2018; Chang *et al*., 2019; Kim *et al*., 2019; Mehrafza *et al*., 2019; Nazari *et al*., 2020).

## MATERIALS AND METHODS

### Study design

This randomized, double-blind, single-arm, controlled clinical trial aimed at evaluating the pregnancy outcomes of infertile women with RIF who had undergone PRP, with RCT: (registered at Iranian Registry of Clinical Trials: N22017073034422), carried out at the Reproductive Biotechnology Research Center, Avicenna Research Institute, ACECR, Tehran, Iran. The ethical committee of the Avicenna Research Institute approved the study and all participants signed and informed written consent.

Among the patients, 214 women who were candidates for frozen-thawed embryo transfer (FET) and had previously failed to conceive after three or more ET with high-quality embryos were assessed for eligibility to enter the study; Among those, 114 women were excluded due to not having the inclusion criteria and consequently, we had 100 women in the study, and we divided them randomly into one of the intervention or control subgroups; thus, the sample size was considered as 100. The inclusion criteria were infertile women with a history of RIF who had failed to achieve a clinical pregnancy despite having at least four good- quality embryos transferred, and were now candidates for IVF/ICSI or freeze embryo transfer cycles with and without the intrauterine infusion of PRP; age below 40 years; and body mass index (BMI) below 30kg/m^2^. The exclusion criteria were hematological and immunological disorders; cancers; hormonal disorders; Hb<11g/dl & PLT<150000mm^3^; chromosomal and genetic abnormalities; taking anticoagulants; taking NSAIDs for 7 days prior to the procedure; smoking; uterine abnormalities (acquired or congenital); FSH <12IU; uncontrolled underlying diseases such as diabetes, and hypertension; simultaneous administration of other drugs (prednisolone, IVIG, GCSF); and simultaneous endometrial scratch.

The trial began by performing hysteroscopy examination, the karyotype of the couples, and laboratory assessment of thrombophilia, antiphospholipid antibodies, as well as hormonal, hematological, and immunological disorders. All participants underwent and FET cycle and were started on estradiol valerate (Aburaihan Co., Tehran, Iran) 6 mg/d was from the 2^nd^ or 3^rd^ day of the menstrual cycle for endometrial preparation and the drug dosage could have been increased to 8mg/d until endometrial thickness reached at least 8 mm on the 10^th^ day of the cycle. The route of administration for estradiol valerate was oral. On the 21^st^ day of the cycle, Diphereline^®^ (vials 3.75 mg intramuscular) (Ipsen/ France), a Gonadotrophin Releasing Hormone analogue (GnRHa) was used for ovary suppression, which was injected intramuscularly. Once the endometrial thickness was 8 mm, progesterone 50 mg (Iran Hormone) was injected intramuscularly for 4 days. Subsequently, good quality embryos (Grade A or B based on embryological grade) were transferred by an Infertility Fellow on the fifth day. PRP was performed for the intervention group patients 48 hours prior to embryo transfer. Both PRP injection into the uterus and embryo transfer were performed under ultrasound guidance. Oral Estradiol valerate and progesterone (Injection-IM) were continued for 2 weeks after ET and in case of having a serum Beta-Human Chorionic Gonadotropin (ß-HCG) positive; those supplementations were continued until 12 weeks of pregnancy.

For PRP, 8.5cc blood was taken from a patient's peripheral vein, the platelet count was checked, and then it was made by using a two-step centrifuging process. An anticoagulant, Acid Citrate 2.5cc (Royagen/ Aria Mabna Tashkhis, Iran) was added and centrifuged for 10 minutes at 1400 rpm to separate the red blood cells. The upper plasma was then separated and centrifuged again at 3500 rpm for another 6 minutes. The platelet count was checked again to ensure a quantity approximately 4-5 times higher than its amount in the peripheral blood. Then 0.5 cc of this solution was injected into the uterine cavity through an IUI transfer catheter (Takwin, Iran). For the control group, only the catheter was transferred and removed without injection or any other action. The control subgroup received routine infertility treatment in the clinic, and no PRP treatment was applied on them. The aim of this study was to evaluate if PRP can be more beneficial to infertility cases than the routine infertility treatments.

### Outcome assessment

Two weeks after transfusion, serum ß-HCG level was measured by ELISA as a sign of embryo implantation, and positive cases were considered successful implantation. In cases of successful implantation, five weeks after ET these patients had a transvaginal ultrasound to see the pregnancy sac and confirm the fetal heartbeat. Cases in which the fetal heart rate was confirmed by ultrasound were considered as clinical pregnancies.

## RESULTS

We had 100 patients with RIF history evaluated in this study, all of whom could complete the study successfully and their data were analyzed afterwards. Four women had a history of hysteroscopic surgery due to Asherman's syndrome and myoma. All participants had a history of almost two failed previous ET attempts. There were six pregnant cases in the intervention group and three pregnant cases in the control group; there was statistically significant difference between the two subgroups. The Fetal heartbeat was seen in all 9 pregnancies and their pregnancies continue. The T-test results proved statistically significant differences between the two groups. The implantation rate was calculated separately for each patient and multiplied by the number of pregnancy sacs with FHR reported in vaginal ultrasound at 4-5 weeks of pregnancy. Table 1 provides the patients' characteristics and the results' summary.

**Table 1.** Summary of patients’ characteristics and outcomes.

Evaluated Items	Intervention group	Control group	*p* value
Age (year)	35	32.7	0.07
BMI (kg/m^2^)	25.3	25.9	0.72
Etiology of Infertility (%)			
Male Factor (MF)	12	10	0.72
Female Factor (*FF*)			
-Anovulation	31	34	0.68
-Tubal Factor	15	10	0.70
-Endometriosis	10	12	0.76
*Mixed* (MF + FF)	42	37	0.64
Diminished ovarian reserve	11	14	0.59
Duration of infertility (year)	4.5	6.5	0.074
The number of transferred embryos (n)	2.8	3.3	0.28
Blood FSH on the third day of cycle (mIU/mL)	7	5.4	0.01
Estradiol intake unit dose (mg)	6.13	6	0.77
Duration of estradiol administration (days)	34	28	0.23
Sperm count (million/ mL)	31.6	34.6	0.9
Normal Sperm morphology (%)	4.07	3.6	0.49
Pregnant cases (n)	20	13.33	0.62
History of previous successful pregnancies (n)	2.7	2.9	0.27

## DISCUSSION

Platelets release granules containing growth factors. More precisely, TGF-ß, PDGF, IGF, VEGF, EGF, and FGF-2 are released from platelets during acute blood loss for repairing vascular walls (Christgau *et al*., 2006) through stimulating the inflammatory cascade and thus the healing process. PRP is a fraction of plasma with concentrated platelets, 4-5 times above the normal range (Marx, 2001). Recently, PRP is widely applied in clinical scenarios for regeneration, even in the eye and mouth (Alio *et al*., 2012). Obviously, due to injecting a single dose of PRP locally, the drug is not thought to cause systemic changes to the immune status of the body or other organs. Local injection of PRP promotes implantation by its increased level of growth factors, increased expression of IL1, IL8, increased progesterone receptors, and proliferation of endometrial cells.

Recurrent implantation failure is still one of the big dilemmas in the field of infertility treatment (Green *et al*., 2015). Although embryonic factors and implantation processes are involved, the endometrium is the critical factor in pregnancy onset and progression (Simon & Laufer, 2012). During the window of implantation, i.e. around days 19-23 of each cycle, a molecular cascade leads to the creation of implantation and pregnancy proteins; cytokines, growth factors, prostaglandins, and adhesion molecules are among these proteins, impairment and mal-expression of these has been proved to be connected with RIF (Granot *et al*., 2012; Simon & Laufer, 2012).

It is hypothesized that since PRP contains various growth factors and cytokines, it could possibly stimulate proliferation and regeneration, enhance endometrial receptivity, and consequently triggers implantation; therefore, local infusion of PRP, which has been collected from an autologous blood sample, and has been enriched with platelets almost 4-5 times higher than peripheral blood, may be beneficial to infertile endometria. A recent study has revealed that embryo transfer cancelation due to thin endometrium could be mitigated by intrauterine infusion of PRP. They realized that all of the five patients who underwent PRP infusion had positive clinical pregnancy. Moreover, the normal continuation of pregnancy was seen in four of the cases (Yu *et al*., 2011).

Since there are many contradictory results in the use of PRP in RIF patients in previous papers, our study aimed at assessing its efficacy in implantation and pregnancy continuity. The result of our study revealed that intrauterine infusion of PRP might have a positive effect on implantation and pregnancy. The rate of chemical and clinical pregnancy in the intervention group was higher than in the control group. There were no side effects recorded in our study. In addition, side effects had not been reported in other studies.

## CONCLUSION

According to our study, PRP may play a role in improving fertility in people with a history of RIF. Our statistical data was limited to 100 patients and we recommend further studies, with larger samples to get definitive results.
